# *In vitro* evaluation of photon and carbon ion radiotherapy in combination with cisplatin in head and neck squamous cell carcinoma cell lines

**DOI:** 10.3389/fonc.2023.896142

**Published:** 2023-04-04

**Authors:** Xumeng Fang, Pian Sun, Yuanli Dong, Yangle Huang, Jiade Jay Lu, Lin Kong

**Affiliations:** ^1^ Department of Radiation Oncology, Shanghai Proton and Heavy Ion Center, Fudan University Cancer Hospital, Shanghai, China; ^2^ Shanghai Key Laboratory of Radiation Oncology (20dz2261000), Shanghai, China; ^3^ Shanghai Engineering Research Center of Proton and Heavy Ion Radiation Therapy, Shanghai, China

**Keywords:** head and neck squamous cell carcinoma (HNSCC), cisplatin, carbon ion radiotherapy (CIRT), relative biological effectiveness (RBE), radiosensitisation

## Abstract

**Background:**

Heavy ion radiotherapy, such as carbon ion radiotherapy (CIRT), has multiple advantages over conventional photon therapy. Cisplatin, as a classic anti-tumor drugs, has been tested and discovered as a photon radiosensitizer in several cell lines, including head and neck squamous cell carcinoma (HNSCC). Hence, the aim of our study is to evaluate whether cisplatin can sensitize CIRT towards HNSCC cell lines *in vitro*.

**Methods:**

Human nasopharyngeal carcinoma cell line CNE-2, human tongue squamous carcinoma cell line TCA 8113 and human hypopharynx squamous carcinoma cell line FADU were all irradiated with photon beam of 2, 4, 6, 8 Gy (physical dose) and carbon ion beam of 1, 2, 3, 4 Gy (physical dose) and treated with cisplatin. Cell survival was assessed by clonogenic survival assay.

**Results:**

CIRT showed significantly stronger cytotoxic effect than standard photon radiotherapy. The relative biological effectiveness (RBE) of carbon ion beam at 10% survival ( $RBE_{10}$ ) was calculated 3.07 for CNE-2, 2.33 for TCA 8113 and 2.36 for FADU. Chemoradiotherapy (both photon radiotherapy and CIRT) was more effective than radiotherapy alone. *In vitro* sensitizer enhancement ratios (SERs) of cisplatin in CNE-2, TCA 8113 and FA DU cell lines after photon irradiation were 1.33, 1.14 and 1.21, while after carbon ion irradiation were 1.02, 1.00 and 0.96, showed that cisplatin sensitized photon irradiation but showed no sensitization effect in carbon ion irradiation in all tested cell lines.

**Conclusions:**

In conclusion, high linear energy transfer (LET) CIRT was more effective than photon irradiation to prevent the proliferation of HNSCC cell lines. Additional treatment with cisplatin could sensitize photon irradiation but showed no effect on carbon ion irradiation.

## Introduction

Head and neck cancers are the seventh most common type of cancer in the world (1). It includes epithelial malignancies that originate from the upper respiratory tract and upper gastrointestinal mucosa that mostly invade the oral cavity, nasal cavity, paranasal sinuses, pharynx, and larynx. Head and neck squamous cell carcinoma (HNSCC) comprises over 90% of head and neck malignancies (2). Therapeutic strategies for HNSCC include surgery, radiotherapy, chemotherapy, targeted therapy and immunotherapy. Because of the complicated anatomical structure and the need to balance patients’ functional retention and the quality of life, multidisciplinary comprehensive treatment (MDT) is essential (3).

With the continuously progress in HNSCC research, concurrent chemoradiotherapy has gradually shown advantages over radiotherapy alone by improving survival in patients with resectable and nonresectable, advanced disease (4, 5). Among all those chemotherapeutic agents, cisplatin-based regimens have been identified as the standard first-line treatment for HNSCC (6). Cisplatin was FDA-approved in 1978 as the first platinum compound for cancer treatment. Investigations have shown that cisplatin is capable of crosslinking with purine bases on DNA, interfering with DNA repair mechanisms, causing DNA damage, adducting formation and subsequently inducing apoptosis of cancer cells (7–9). Cisplatin, as a classic anti-tumor drugs, has been tested and discovered as a radiosensitizer in several cell lines (10, 11). Indeed, numerous trials have demonstrated that concurrent single-agent cisplatin chemoradiotherapy significantly improves the survival of HNSCC patients compared to radiotherapy alone (12–14).

Heavy ion radiotherapy, such as through the use of carbon ion radiotherapy (CIRT), has multiple advantages over conventional photon therapy. CIRT can spare surrounding normal tissues while killing tumors due to its superior physical dose distribution (i.e. Bragg Peak). As a high-LET radiation, CIRT mainly breaks DNA double-strands and damages tumor cells, while it is not affected by cell cycle and oxygen concentration. Therefore, CIRT is effective against tumors that are resistant to hypoxia and photon radiation (15).

The sensitization of chemotherapy to carbon ions in tumor cell lines has been investigated for multiple chemotherapeutic agents (e.g. temozolomide, gemcitabine, cisplatin, camptothecin and paclitaxel) and tumor cell lines (e.g. glioblastoma, pancreatic cancer, esophageal squamous cell carcinoma and colorectal tumor). In clinical practice, results of clinical trials of the National Institute of Radiological Sciences (NIRS) showed that the combination of CIRT and gemcitabine treatment is not superior to CIRT alone in treating locally advanced pancreatic cancer (16).

The use of concurrent chemotherapy plus high-LET irradiation, such as CIRT, for radiosensitization has never been addressed in HNSCC. Therefore, this study aims to evaluate the cytotoxic effect of photon and carbon ion radiotherapy in combination with cisplatin in HNSCC cell lines, aiming to improve current and future clinical practice.

## Materials and methods

### Cell lines and culture conditions

The human nasopharyngeal carcinoma cell line CNE-2 was obtained from the Xiangya Hospital, Central South University, Changsha, Hunan, China, and was cultured in RPMI 1640 supplemented with 10% heat-inactivated fetal calf serum (Gibco, USA), 1% penicillin/streptomycin (Gibco, USA), at 37 °C in a humidified atmosphere containing 5% CO2.

The human tongue squamous carcinoma cell line TCA 8113 and the human hypopharynx squamous carcinoma cell line FADU were purchased from Shanghai Zhong Qiao Xin Zhou Biotechnology. TCA 8113 was cultured in Dulbecco’s modified Eagle’s medium (DMEM) supplemented with 10% heat-inactivated fetal calf serum (Gibco, USA), 1% penicillin/streptomycin, at 37 °C in a humidified atmosphere containing 5% CO2, while FADU was cultured in minimum Eagle’s medium (MEM) supplemented with 10% heat-inactivated fetal calf serum (Gibco, USA), 1% penicillin/streptomycin, at 37 °C in a humidified atmosphere containing 5% CO2.

### Photon radiotherapy

Cells in the log-growth phase were plated in T-25 flasks (Corning, NY, USA) and irradiated with a 225 kVp X-ray beam (PXi precision X-RAD 225, dose rate: 3.198 Gy/min, 225 kV, 13.3 mA, 40 cm SSD, LET: ~2 keV/μm) at room temperature. Photon radiotherapy was performed as a single exposure to physical doses of 2 Gy, 4 Gy, 6 Gy or 8 Gy.

### Carbon ion radiotherapy

For CIRT, cells in the log-growth phase were plated in T-25 flasks (Corning, NY, USA) and irradiated with the carbon ion beam at room temperature by an IONTRIS intensity-modulated raster scan system with an energy of 333.82 Mev/u at the Shanghai Proton and Heavy Ion Center (SPHIC, Shanghai, China). The LET was approximately 29.1351 keV/μm. The homogeneous spread-out Bragg peak (SOBP) of the carbon ion beam was adjusted to the surface where the adherent cells attached. CIRT was performed as a single exposure to physical doses of 1 Gy, 2 Gy, 3 Gy or 4 Gy.

### Treatment with cisplatin

All cell lines were incubated with cisplatin (Sigma-Aldrich, Germany) for 4 hours, followed by a medium change and exposure to radiation (photon irradiation or carbon ion irradiation). Pre-experiments were performed in 6-well cell culture plates to determine the experimental concentration of cisplatin. Cells were incubated with 1, 2, 3, 5, 10 or 20μM cisplatin for 4 hours and irradiated with 4 Gy or 6 Gy photon radiotherapy. Doses were selected to allow 50~60% cell survival after combination therapy confirmed by microscopic inspection, to keep enough cells alive when they were subjected to carbon ion radiotherapy combined with cisplatin chemotherapy. After pre-experiments the following drug concentrations were chosen: 3μM for CNE-2, 1μM for TCA 8113 and 2μM for FADU.

### Clonogenic survival assay and statistical analysis

Clonogenic survival assay, as the radiobiological gold standard, was performed to assess the response of cell lines to radiation and chemotherapy. Cells (10^3^ to 10^4^) were seeded in 25 cm² flasks (Corning, NY, USA). After cell attachment, which was confirmed by microscopic inspection, cells were exposed to a 4-hour chemotherapeutic incubation and were irradiated immediately after cisplatin was removed. After 7-14 days, single cells grew into colonies with over 50 cells, then they were gently washed with phosphate buffer saline (PBS) (Beyotime Biotechnology, Shanghai, China) twice, fixed with 4% paraformaldehyde for 30 minutes, gently washed with PBS twice again, and stained with crystal violet (Beyotime Biotechnology, Shanghai, China) for 30 minutes. Upon a final wash, colonies were counted by a colony counter (Oxford Optronix, UK) to detect colonies with over 50 cells. There were four groups: X-ray only group, X-ray+cisplatin group, Carbon ion only group, and Carbon ion+cisplatin group. For each cell line, data were obtained by three independent experiments and each experiment was run in triplicates.

Clonogenic survival curves were generated through GraphPad Prism version 7.00 (GraphPad Software, San Diego California, USA) according to cell plating efficiency and clonogenic survival. Cell survival data were fitted by the linear quadratic (LQ) model: S  = exp (- α D- $\beta D^{2}$ ), where S is the survival fraction, D is the dose in Gray, α (G $y^{-1}$ ) is the single-hit inactivation coefficient and β (G $y^{-2}$ ) is the maximal double-hit inactivation coefficient (no repair). Cell survival data were also estimated by a multitarget single-hit model: 


$$S=1-\;\left(1-e^{-\frac{D}{D0}}\right)N,$$

where S is the survival fraction, D is the dose in Gray, D_0_ is the mean lethal dose, which is calculated from the reciprocal of the slope of the survival curve, that is,


$$\;D_{0}=1/k$$

Sensitizer enhancement ratios (SERs) were calculated as the ratio of the D_0_ of cells treated with irradiation alone to the D_0_ of cells treated with irradiation plus another treatment, which was used to quantify the sensitization effect of radiotherapy. Significant differences among different groups were determined by unpaired Student’s t-test with a 2-tailed distribution. P value<0.05 was considered statistically significant.

## Results and discussion

### Results

#### Radiobiological parameters and evaluation of the cytotoxic of photon and carbon ion radiotherapies

From linear quadratic fits (LQ-fits), several radiobiological parameters for photon and carbon ion radiotherapies were determined in three cell lines (see **Table 1**).

**Table 1:** Data were fitted to the linear quadratic model for X-ray and carbon ion irradiation in CNE-2, TCA 8113, and FADU cell lines, and the indicated radiobiological parameters were determined from the fitted curve.

Radiobiological parameters	CNE-2		TCA 8113		FADU	
	X-ray	Carbon ion	X-ray	Carbon ion	X-ray	Carbon ion
α	0.2099	0.3384	0.2238	0.7325	0.2269	0.5727
β	0.01476	0.2689	0.04888	0.1662	0.1775	0.9597
α/β	14.221	1.258	4.579	4.407	1.278	0.597

α (Gy^ (-1)) is the single-hit inactivation coefficient; β (Gy^ (-2)) is the maximal double-hit inactivation coefficient (no repair).

The concept of RBE has been used to describe the efficiency of different types of radiation to produce biological effects. It’s defined as the ratio of a dose from the reference radiation, photons, to a dose from any other radiation quality to produce the same biological effect. The RBE of carbon ion beams at 10% and 37 % survival for each cell line was calculated from the LQ-fits to evaluate the advantages of carbon ion radiotherapy over photon radiotherapy (see **Table 2**).

**Table 2:** *RBE-* relative biological effectiveness*;* $RBE_{10}$ *- the RBE at 10% survival level;* $RBE_{37}$ *- the RBE at 37% survival level*.

Cell line	CNE-2	TCA 8113	FADU
RBE_10_	3.072	2.331	2.360
RBE_37_	2.689	2.543	2.378

#### Evaluation of cytotoxic effect of chemoradiotherapy

**Figure 1:**
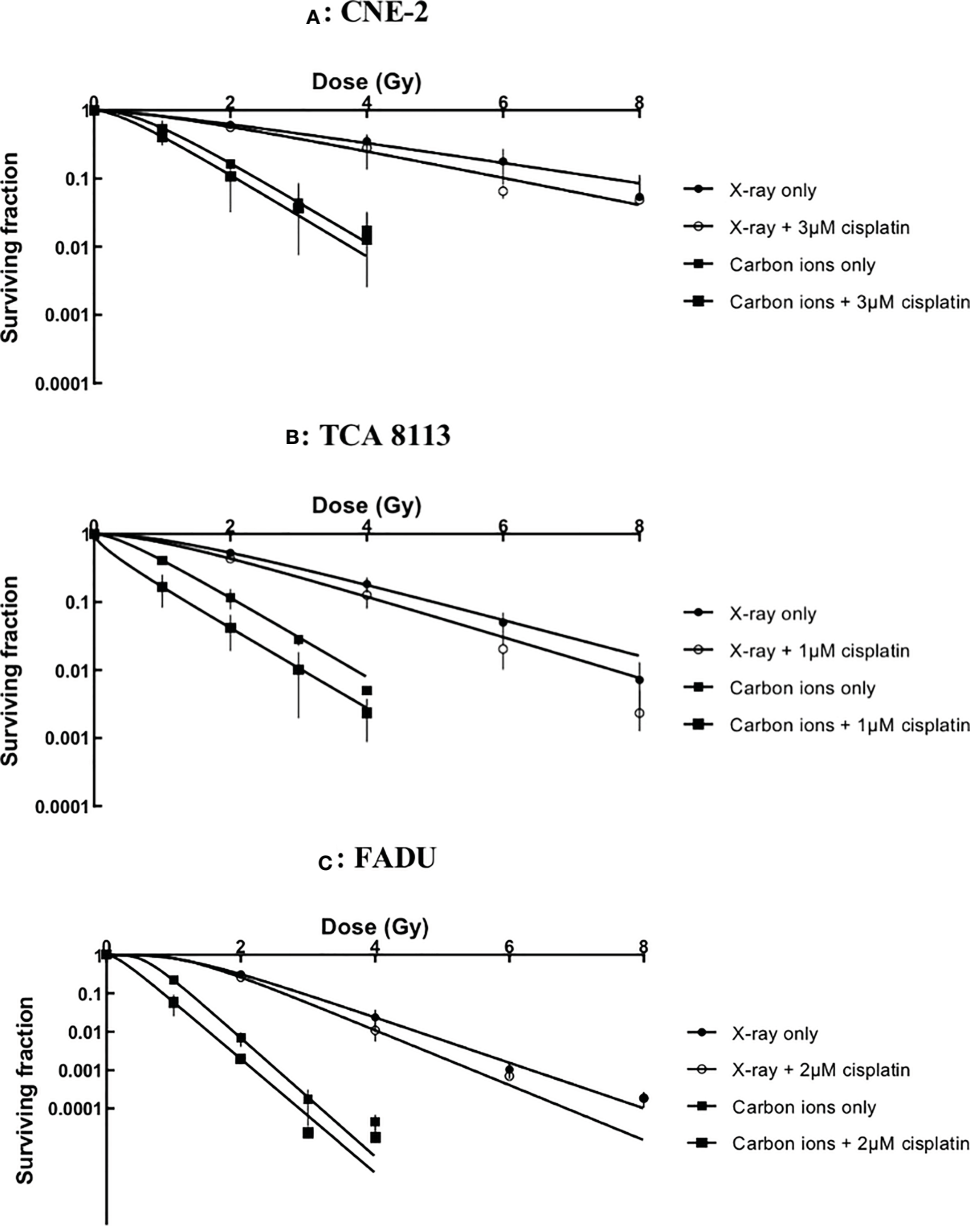
Survival curves of chemoradiotherapy experiments in CNE-2 **(A)**, TCA 8113 **(B)** and FADU **(C)** cell lines.

The cytotoxicity of cisplatin with radiation was assessed by the clonogenic survival assay. Survival data were fit to a multitarget-single hit model to construct survival curves (see **Figure 1**).

Cisplatin plus radiotherapy was more effective than radiotherapy alone in every cell line. However, further calculations were necessary to accurately evaluate the impact of cisplatin on X-ray and carbon ion irradiation.

SERs were used to indicate radiosensitization and were calculated to evaluate the cytotoxic effect of photon and carbon ion radiotherapies in combination with cisplatin. According to previous studies, SERs were mostly calculated as the ratio of the D_0_ of cells treated with irradiation alone to the D_0_ of cells treated with irradiation plus another treatment. In our case, SERs values ranged from 0.96-1.33 depending on the cell line and applied irradiation (see **Table 3**). In the CNE-2 cell line, cisplatin markedly sensitized photon irradiation but did not affect carbon ion irradiation. In TCA 8113 cells, cisplatin slightly sensitized photon irradiation but showed no sensitization effect to carbon ion irradiation. Finally, in FADU cells, cisplatin resulted in an obvious sensitization for photon irradiation but not for carbon ion irradiation. Overall, cisplatin sensitized photon irradiation but showed no sensitization effect in carbon ion irradiation in different HNSCC cell lines.

**Table 3:** SER_D0_ values of three HNSCC cell lines.

Cell line	CNE-2		TCA 8113		FADU	
	D_0_	SER_D0_	D_0_	SER_D0_	D_0_	SER_D0_
X-ray only	2.862	–	1.636	–	0.734	–
X-ray+cisplatin	2.160	1.33	1.440	1.14	0.607	1.21
Carbon ion only	0.735	–	0.742	–	0.282	–
Carbon ion+cisplatin	0.723	1.02	0.743	1.00	0.294	0.96

SERs were calculated as the ratio of the D0 of cells treated with irradiation alone to the D0 of cells treated with irradiation plus another treatment.

### Discussion

The present study demonstrated that CIRT is more potent at killing HNSCC cells than conventional photon irradiation. This is in line with the current understanding of CIRT since previous studies have shown that CIRT is able to counteract migration and invasion of HNSCC parental cells and cancer stem cells (CSCs) (17). Moreover, high LET irradiation can also induce distinct types of cell death in HNSCC cell lines (18), indicating that CIRT is a more effective therapeutic modality than photon irradiation, which is consistent with our experimental results.

In order to quantify the advantages of CIRT over conventional photon radiotherapy, researchers have consistently used RBE values to evaluate effects. RBE is a crucial indicator to compare the biological effects of different types of radiation since it is closely related to the determination of radiation dose, while it is a complex parameter that depends on both physical (e.g. LET, dose rate, particle type and energy) and biological (e.g. cell type, cell condition, tissue type, cell cycle phase and oxygen condition) parameters. In our study, the RBE_10_ and RBE_37_ were respectively: 3.07 and 2.29 for CNE-2, 2.33 and 2.54 for TCA 8113, and 2.36 and 2.38 for FADU. Therefore, the RBE for CIRT was calculated to be between 2.33 and 3.07 for HNSCC cell lines. These results can assist subsequent research on CIRT.

We evaluated whether cisplatin could act as a radiosensitizer for radiotherapy in HNSCC cell lines. In several previous studies, and in this one, SER was calculated as the ratio between D_0_ of cells treated with irradiation alone and D_0_ of cells treated with irradiation plus another treatment (19–21). In contrast, some studies used different comparison methods, such as comparing D_10_ (22), D_50_ (23) and SF values (24). Comparison of D_0_ values may be more informative since this is the only parameter that can reflect the state of the entire survival curve. As mentioned before, D_0_ was calculated from the reciprocal of the slope of the survival curve, however, other parameters (such as D_10_, D_50_ and SF values) can only reflect a certain part of the survival curve, therefore their reference value is lower than that of D_0_.

Our study has focused on the evaluation of whether cisplatin could act as a radiosensitizer for CIRT in HNSCC cell lines. Cisplatin has been reported as a radiosensitizer in several cell lines, including human non-small cell lung cancer cell line A549 (19, 25, 26), human cervical cancer cell line CaSki (26), rat hepatoma cell line H4 (27) and mouse fibrosarcoma cell line RIF1 (10). However, cytological studies investigating the radiosensitization effect of cisplatin for photon irradiation in HNSCC cells are still lacking. There is one *in vitro* study that used FADU cells and compared D_50_ (dose with 50% cell survival) values (23). If this comparison method were to be used in the data herein contained, the D_50_ value of the X-ray-only group was 1.44, while that of the X-ray plus cisplatin group was 1.33, that is, the ratio between the two treatments was approximately 1.1, which is consistent with this previous study (23). In 2015, Ziemann et al. reported that among eight HNSCC cell lines, including four HPV-positive (HPV+) (UM-SCC-47, UM-SCC-104, 93-VU-147T, UPCI:SCC152) and four HPV-negative (HPV-) (UD-SCC-1, UM-SCC-6, UM-SCC-11b, UT-SCC-33) cell lines, combined treatment with cisplatin and X-ray irradiation led to an enhanced cytotoxic effect in all cell lines except in UD-SCC-1. The radiosensitizing effect of cisplatin was more pronounced in HPV+ cells compared to that of HPV- cell lines. Similar results were found in two other studies (23, 28). Therefore, cisplatin showed radiosensitizing effects for photon irradiation on almost all HNSCC cell lines tested. The mechanism for the radiosensitivity toward photon radiotherapy has been investigated and includes the interaction between cisplatin-induced DNA adducts and radiation-induced strand breaks (29), apoptosis induction and cellular senescence (30).

The sensitization of chemotherapy to carbon ions in tumor cell lines has been shown to be related to the chemotherapeutic agent and tumor type. Combs et al. and Harrabi et al. reported that temozolomide showed no radiosensitizing effect on CIRT for glioblastoma cells (31, 32), which shared the same chemotherapeutic agent and tumor type. Sai et al. reported that gemcitabine could sensitize CIRT in pancreatic cancer stem-like cells (33), while Kitabayashi et al. reported that gemcitabine showed no effect on esophageal squamous cell carcinoma (34). Meanwhile, Harrabi et al. reported that gemcitabine could sensitize CIRT in colorectal tumor cell lines (35), whereas Schlaich et al. reported that cisplatin showed no radiosensitizing effect for colorectal tumor cell lines (36).

Focusing on cisplatin, Sai et al. reported that cisplatin sensitized CIRT in triple-negative breast cancer stem-like cells (37) and malignant mesothelioma cell lines (38), but other studies indicated that cisplatin showed no radiosensitizing effect for glioblastoma (39), esophageal squamous cell carcinoma (34) and colorectal tumor cell lines (36). Our study is the first cytological experiment to investigate if cisplatin is a radiosensitizer for CIRT in HNSCC cells. According to SERs of cisplatin to CIRT there was no radiosensitizing effect exerted by cisplatin in HNSCC cell lines. Several mechanisms may explain why cisplatin does not sensitize CIRT, despite a clear sensitization of photon irradiation. These mechanisms include cell apoptosis and senescence induced by carbon ion irradiation, X-ray irradiation and cisplatin treatment, and mitotic catastrophe that is only triggered by carbon ions. In this regard, mitotic catastrophe is a putative mechanism underlying the weak correlation between sensitivity to carbon ions and cisplatin (30).

Our study was performed in preparation for further clinical trials to investigate the potential of CIRT in the treatment of HNSCC. Clinical evidence shows that the addition of cisplatin increases the efficacy of X-ray radiotherapy toward patients with cervical cancer (40) and non-small-cell lung cancer (41), as well as patients with HNSCC (42–44), which are consistent with our results. However, to the best of our knowledge, there is no clinical study focusing on CIRT plus cisplatin to treat HNSCC. Okonogi et al., who worked at the Heavy Ion Medical Accelerator in Chiba (HIMAC) at the NIRS, reported a phase I/ II clinical trial for patients with locally advanced uterine cervical adenocarcinoma treated with CIRT (74.4 GyE/20 fractions) and cisplatin (40 mg/m^2^/week). The 2-year LC of this study was 71%, and the 2-year OS was 88% (45). This group also reported a phase I/II clinical trial for patients with locally advanced uterine cervical adenocarcinoma treated with single CIRT (72.0~72.8 GyE/20 fractions and 64.0~68.8 GyE/20 fractions). The 5-year LC was 72%, the 5-year OS was 47%, the 10-year LC was 72% and the 10-year OS was 39% (46). Unfortunately, the doses of CIRT in these two trials were different, and the published evaluation criteria of clinical outcomes were also different, therefore it is difficult to conclude from the above data whether cisplatin can sensitize carbon ions in locally advanced uterine cervical adenocarcinoma. Interestingly, Mizoe et al. reported a phase II clinical trial for patients with head and neck cancers, in which the patients were treated with CIRT (64.0 GyE/16 fractions) at the NIRS. This study concluded that the 5-year local control rate of HNSCC was 61% and the 5-year overall survival rate was 17% (47). Therefore, if we can assure that patients will not develop serious side effects, clinical studies of carbon ions in combination with cisplatin may be performed in the future.

## Conclusions

In conclusion, high LET CIRT was more effective than photon irradiation to prevent the proliferation of HNSCC cell lines. Additional treatment with cisplatin could sensitize photon irradiation but showed no effect on carbon ion irradiation. This study can provide information for current and future clinical trials and clinical practice.

## Data availability statement

The raw data supporting the conclusions of this article will be made available by the authors, without undue reservation.

## Author contributions

XF designed experiments, performed experiments, arranged the data, did statistical analysis and wrote the manuscript. All authors contributed to the article and approved the submitted version.
